# Knowing What to Respond in the Future Does Not Cancel the Influence of Past Events

**DOI:** 10.1371/journal.pone.0005607

**Published:** 2009-05-29

**Authors:** Elisabet Tubau, Joan López-Moliner

**Affiliations:** Visió i Control de l'Acció, Departament de Psicologia Bàsica, Facultat de Psicologia, Universitat de Barcelona, Barcelona, Catalonia, Spain; University of Granada, Spain

## Abstract

Everyday tasks seldom involve isolate actions but sequences of them. We can see whether previous actions influence the current one by exploring the response time to controlled sequences of stimuli. Specifically, depending on the response-stimulus temporal interval (RSI), different mechanisms have been proposed to explain sequential effects in two-choice serial response tasks. Whereas an automatic facilitation mechanism is thought to produce a benefit for response repetitions at short RSIs, subjective expectancies are considered to replace the automatic facilitation at longer RSIs, producing a cost-benefit pattern: repetitions are faster after other repetitions but they are slower after alternations. However, there is not direct evidence showing the impact of subjective expectancies on sequential effects. By using a fixed sequence, the results of the reported experiment showed that the repetition effect was enhanced in participants who acquired complete knowledge of the order. Nevertheless, a similar cost-benefit pattern was observed in all participants and in all learning blocks. Therefore, results of the experiment suggest that sequential effects, including the cost-benefit pattern, are the consequence of automatic mechanisms which operate independently of (and simultaneously with) explicit knowledge of the sequence or other subjective expectancies.

## Introduction

We unfold our actions within the context of other actions and often we need to execute our action responses very fast. We know that responses to series of stimuli presented at fast rates are affected by the previous stimulus-response events (see [1] for a review). According to the temporal interval between the response and the next stimulus (RSI) and to the number of previous events that are considered, different sequential effects can be observed. For example, in two-choice serial response tasks with short RSI, it is commonly observed a first-order repetition effect: response repetitions are faster than response alternations. Simultaneously, a higher-order repetition effect also emerges: responses, either repetitions or alternations, are faster after repetitions [2]–[5]. Whereas the first-order repetition effect is supposed to rely on an automatic facilitation mechanism, based on the memory trace of the previous stimulus-response event [3], [4], the higher-order repetition effect is considered to reflect response-monitoring activity, which would be higher for response alternations [6]. As memory traces attenuate with time [2], [6], the benefit for repetitions usually vanishes at RSIs longer than 300 ms till the point that a repeating cost can emerge, especially when spatial dimensions are involved (spatial responses corresponding to the stimulus location; [4], [6]). However, when several previous events are considered, a cost-benefit pattern is observed: response alternations are faster after other alternations but they are slower after repetitions. Similarly, repetitions are faster after other repetitions but they are slower after alternations.

Unlike the sequential effects at short RSIs, sequential effects observed at long RSIs are usually considered to be caused by subjective expectancies [3], [4]. Subjective expectancies are commonly defined as strategic, top-down influences on motor reaction-times [6]–[8]. Based on the distinction between passive or automatic expectancies and active or conscious expectancies [9], sequential effects observed at long RSIs are thought to be caused by active variants of expectancy [10], [11]. Subjective expectancies are considered to be active because they take longer to develop than the passive ones [7] and because only active expectancies are thought to produce benefits for expected events and costs for unexpected ones. That is, as suggested by [9], passive or automatic expectancies produce processing benefits for expected events but no costs for unexpected ones whereas active expectancies produce both benefits and costs. Specifically, active expectancies in two-choice random tasks have been often related to probabilistic fallacies as the gamblers fallacy (the irrational belief that alternations are more frequent than repetitions in random series of two, equally likely events [3], [8]) and the continuation of the run fallacy (the irrational belief that local regularities, runs of either repetitions or alternations, tend to continue [7]). See also [12] for an introduction to these probabilistic fallacies.

Nevertheless, it remains unclear the extent to which sequential effects at long RSIs are really caused by the subjective expectancy hold by the participant. Some previous studies have analyzed the impact of induced expectancies on sequential effects [11], [13]. These studies reported that first-order sequential effects were not modulated by such expectancies. Nevertheless, expectancies were induced by means of instruction (i.e. asking participants to expect either a repetition or an alternation) but participants knew that alternations and repetitions were equally likely; so it was possible that participants were holding different expectancies than the experimentally intended ones. On the other hand, expectancies have been manipulated in the context of a sequence learning task [14]. However, in Soetens et als experiment only the stimuli followed a probabilistic sequential pattern, being both responses equally likely. Therefore, regarding the forthcoming stimulus-response event, it was impossible to infer which expectancies participants were holding. In a different but analogous paradigm, Perruchet et al. [15] tried to discover the participants running expectancy by asking them to make a judgment in each trial. In their task, either tones alone or tones followed by a visual stimulus (a square) were presented randomly. Participants had to respond only in the latter case but always had to judge, before each trial, the likelihood of the square being shown after the tone. Motor responses were faster after several tone-square trials (in which the response was required) and slower after several tone-alone trials (in which the response had to be inhibited). In contrast, and according to the gamblers fallacy, participants conscious expectancy of the square increased with the length of the run of tone-alone trials and decreased with the length of the run of tone-square trials. Interestingly, the cost-benefit pattern observed in two-choice tasks may also reflect a dissociation from active expectancies if the gamblers fallacy held; whereas the gamblers fallacy would predict an alternation after a long run of repetitions, it is, indeed, slower. Nevertheless, asking the participants to report expectancies while performing speeded responses makes matters more complicated.

In order to analyze the impact of active expectancies on sequential effects, we used a fixed sequence learning task. If participants were able to acquire complete knowledge of the sequence, a reduction of the response costs should be observed because all the events would be correctly expected. However, if sequential effects as the cost-benefit pattern were inevitable, they would be observed regardless of the ongoing subjective expectancy. Furthermore, according to the level of knowledge acquired, the sequence learning task allows identifying types of learners, making the comparison between participants holding different subjective expectancies possible. In previous experiments with a fixed two-choice sequence, we found that knowledge modulated first-order sequential effects [16], [17]. Whereas participants who did not learn the complete order responded faster to alternations than to repetitions, no differences between transitions were observed in the case of good learners. Unfortunately, analyses of higher-order sequential effects were impossible as no repetition after another repetition was presented (the repeating pattern was RLRRLLRL R = Right, L = Left). Hence, to analyze if cost-benefit patterns would appear regardless of knowledge, a sequence with longer runs of repetitions was used in the present experiment. If, as has been often suggested, the cost-benefit pattern was the consequence of probabilistic fallacies, precise knowledge of the order would attenuate or change this pattern. However, if the cost-benefit pattern was caused by automatic mechanisms, it should be observed regardless of the acquired knowledge.

## Results

Each trial was classified according to the first-order (FO repetition and FO alternation) and to the second-order transitions (SO repetition and SO alternation). Hence, for example, response to the last right stimulus in the pattern left-right-right was coded as a first-order repetition (FO-R) and a second-order alternation (SO-A). We also created the post-hoc factor Knowledge by assigning participants to the Explicit group when they reproduced, at least once, the complete sequence (elements and order) correctly and to the Non-explicit otherwise. As the sequence was presented continuously within a block, any starting point was considered correct (e.g. RLRLRRRLLL; RRRLLLRLRL; RLRRRLLLRL). Percentages of Explicit learners were 35% (8 out of 23) and 50% (7 out of 14) for short and long RSI conditions, respectively. Trials with errors (2% in the explicit group and 5% in the non-explicit group), following errors, and trials with RTs greater than 1000 ms were dropped. The RT ANOVA with block (4 learning blocks), FO (first-order repetitions and alternations) and SO (second-order repetitions and alternations) as within-participant variables and RSI (short and long) and Knowledge (Explicit and Non-explicit) as between ones, showed the following effects:

### Learning effects

**Figure 1:**
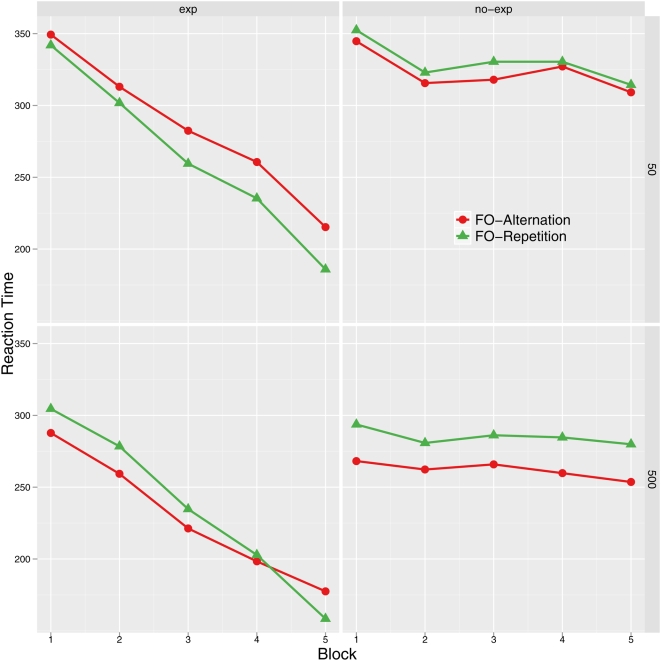
Reaction time means in milliseconds for each block, split by first-order transition. Top: long-RSI condition. Bottom: short-RSI condition. Right: Non-explicit groups. Left: Explicit groups (R - random block).

Both the effects of block and Knowledge were significant (F(3,99) = 39.46 and F(1,33) = 26.92, respectively, ps<.001) as well as the block×Knowledge interaction (F(3,99) = 18.69, p<.001). Explicit learners responded faster than Non-explicit ones and block was only reliable in the former group (F(3,39) = 30.01, p<.001), reflecting that only explicit learners responded increasingly faster. Indeed, explicit learners anticipated almost all the stimulus-response events in the last learning block (see Figure 1).

### Sequential effects

**Figure 2:**
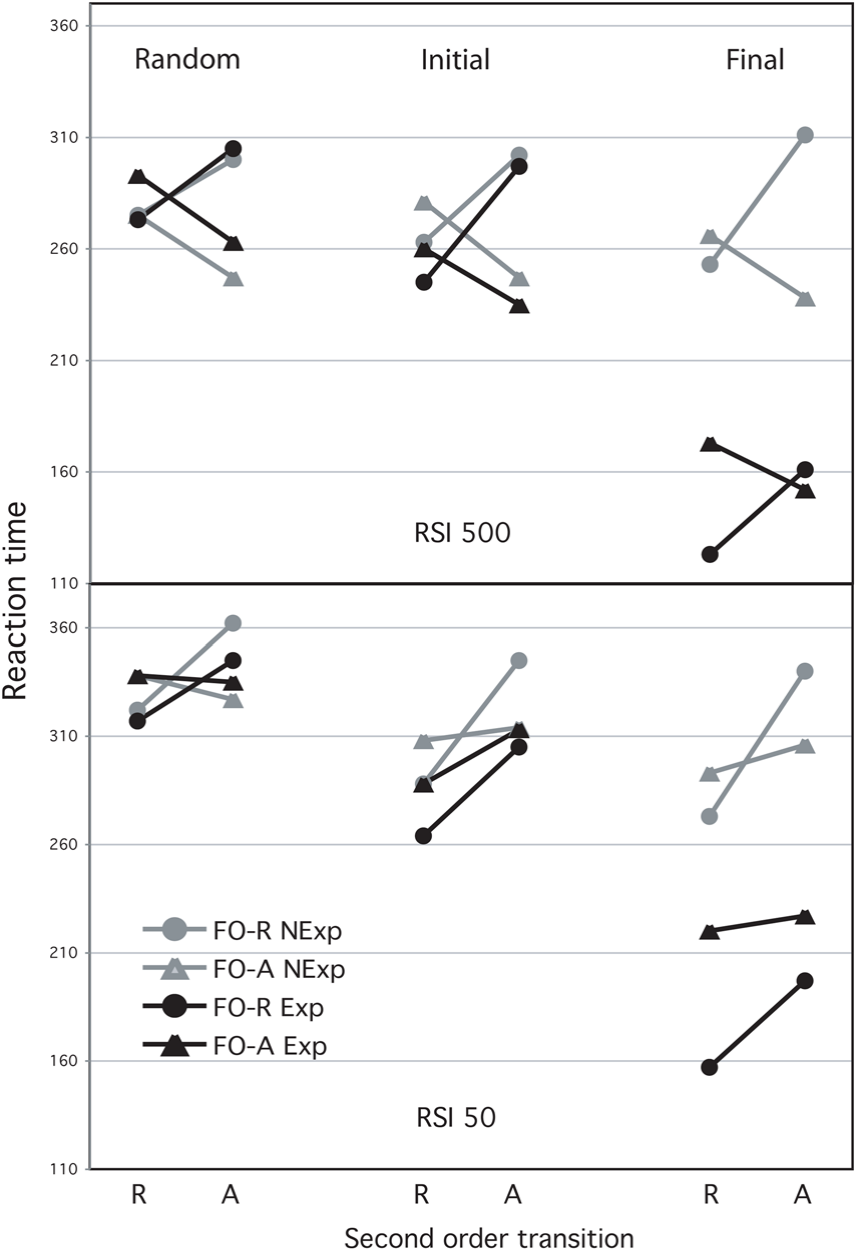
Reaction time means in milliseconds after each second-order transition, split by first-order transition (FO; R: repetition A: Alternation) and Knowledge-group (Explicit and Non-Explicit) in the initial random block (Random) in the initial sequence block (Initial) and in the final sequence block (Final). Top: long-RSI condition. Bottom: short-RSI condition.

The effects of SO and RSI were also significant (F(1,33) = 30.48, p<.001 and F(1,33) = 10.42, p<.01, respectively). Globally, the long RSI condition produced faster responses than the short RSI one and responses after a repetition were faster than after an alternation. RSI interacted with FO (F(1,33) = 7.27, p<.01), with SO (F(1,33) = 9.11, p<.01) and with FO×SO (F(1,33) = 7.37, p<.01). FO was only significant in the long RSI condition (F(1,12) = 5.83, p<.05), reflecting a significant first-order alternation effect (see Figure 1). SO was reliable in both RSI conditions, reflecting a global second-order repetition effect (see Figure 2). However, it was stronger in the short RSI one (F(1,21) = 32.79, p<.01, and F(1,12) = 8.01, p<.05; for short and long RSI conditions, respectively). Also, the FO×SO interaction was reliable in both conditions, although it was stronger in the long RSI one (F(1,21) = 16.99, p<.01, and F(1,12) = 68.88, p<.001; for short and long RSI conditions, respectively; see Figure 2). Hence, the cost-benefit pattern was reliable in both RSI conditions and in both groups of learners (see below).

### The impact of explicit knowledge on sequential effects

Interestingly, Knowledge interacted with FO (F(1,33) = 10.59, p<.01) and the triple Knowledge×block×FO interaction was also significant (F(3,99) = 6.11, p<.01). FO was only significant in the case of the Non-explicit group (F(1,20) = 7.91, p<.05) and the block×FO interaction was only reliable in the Explicit group F(3,39) = 5.95, p<.05). Whereas Non-explicit learners were faster on alternations than on repetitions, a repetition effect emerged in the last learning block in the case of the Explicit group (F(1,13) = 12.05, p<.01). This effect tended to be earlier in the short RSI condition, though the triple RSI×FO×Knowledge was not significant (see Figure 1). Crucially, the Knowledge factor did not interact neither with SO nor with SO×FO (all Fs<1), reflecting that second-order sequential effects were equivalent, regardless of the type of expectancy (see Figure 2).

### Random serial block

The RT ANOVA with the initial random block showed similar patterns. RSI×FO and RSI×SO were significant (F(1,33) = 5.98, F(1,33) = 5.26, respectively, ps<.05). FO was only significant in the long RSI condition (F(1,12) = 13.27, p<.01), showing a global alternation effect. Differing from the learning blocks, SO was significant only in the short-RSI condition (F(1,21) = 12.32, p<.01); reflecting a significant second-order repetition effect in this condition. The FO×SO interaction was globally significant (F(1,33) = 46.38, p<.001) and it did not interact with RSI (see Figure 2).

## Discussion

In line with previous studies on sequence learning [16], [18], [19] results of the reported experiment showed a high correlation between performance and explicit knowledge. Participants who acquired complete knowledge of the order responded faster and more accurately than non-explicit learners. The effect of knowledge was clearly demonstrated by the tendency of explicit learners to make correct anticipations in the final learning blocks. In spite of this anticipatory activity, second-order sequential dependencies were observed in all the participants. Specifically, the cost-benefit pattern varied neither through learning blocks, nor between groups of learners. As shown in Figure 2, both groups showed facilitation for repetitions after another repetition in both RSI conditions. Furthermore, in the long RSI condition, both groups showed repetition costs after an alternation. Provided that explicit learners were not holding any irrational probabilistic expectancy, such effects should have been caused by more automatic mechanisms. The first-order repetition effect was observed neither in the global RTs analyses nor in the short RSI condition. It could be argued that the fixed pattern was biased towards the alternation response (it contained 60% of alternations), explaining thus the absence of the repetition effect. However, the same result was observed in the initial random block, where both transitions were equally likely. Furthermore, the repetition effect increased through the learning phase in the case of Explicit learners. In line with this finding, the repetition effect has not been always observed [3], [10], [20] suggesting that it may be vulnerable to certain individual differences and/or specific experimental procedures. Furthermore, the experiments showing the repetition effect have usually more trials than in the reported one [4], [5]. In contrast to the delay in the expression of the repetition effect, non-explicit learners showed an alternation effect, together with a cost-benefit pattern, since the initial random block. The alternation bias was also observed in the percentage of errors; 10% of the times in which a repetition was required after an alternation, another alternation was produced. It is worth noting that these erroneous responses, though less frequent in the explicit group (5%), were not totally overcome, suggesting an underlying conflict between the correct explicit prediction for a repetition and the automatic tendency to prepare the alternate response after another alternation. The fact that this tendency was expressed very early (the same pattern was observed in both RSI conditions) and that, regarding RT, had the same impact on both groups of participants, suggests that automatic mechanisms rather than active or strategic expectancies (e.g. the gamblers fallacy: [3], [7], [8]) may be involved. Based on time course constraints, and when spatial stimuli are involved, this mechanism could be related to the inhibition of return phenomenon (see also [21] for a similar proposal). As in the present findings, the inhibition of return changes to a benefit for repetition after a repetition of the same location. Interestingly, using other visuomotor tasks, it has also been shown that previous trial history, rather than knowledge of the future, determines automatic preparation processes [22].

In conclusion, the reported results showed that knowledge of future events does not override the influence of past events, neither at short nor at long RSI. Specifically, memory of the previous pattern (repetition or alternation) had an automatic effect on performance, regardless of the explicit knowledge of the sequence. To this point, these results challenge the idea of a strategic or active nature of the expectancies producing the cost-benefit pattern. Rather, they clearly showed the involvement of automatic pattern detector mechanisms (see also [23]) which, as shown in the present data, operate independently of (and simultaneously with) the acquired knowledge or other subjective expectancies.

## Materials and Methods

### Ethics Statement

Written consent was obtained from each participant prior to the experiment. The experiment was approved by the local ethics committee of the University of Barcelona.

### Participants

A total of 37 students from the University of Barcelona participated as a requisite for extra course credits. Participants were randomly distributed to the long and to the short RSI conditions (14 and 23, respectively; Only a few participants were able to learn the complete sequence in the short RSI condition. Hence, in order to have more participants in the explicit group, we had to increase the sample). The Knowledge factor was created post-hoc from the analyses of the final test given to the participants (see procedure).

### The task

A white X (0,5 cm high) on a black background, was presented either 3.0 cm to the left or to the right from the centre of the screen of a compatible IBM PC. The spatial position of the stimulus corresponded to the left and right responses, which were operated with left and right index fingers, respectively. In the fixed sequence blocks the stimuli were presented according to the repeating pattern introduced above (RLRRRLLLRL; R: Right stimulus-response; L: Left stimulus-response). The stimulus was on the screen till the response. RSI was 50 ms in the short RSI condition and 500 ms in the long one.

### Procedure

Participants were assigned randomly to either the short or long RSI conditions. They were instructed to respond with the button corresponding to the location of the letter X as fast and as accurately as possible. Participants received incidental instructions, that is, the repeating pattern was not mentioned and the experiment was introduced as one of exploring the effect of training on RT. The sequence learning task was formed by 4 blocks of 180 trials each. Brief pauses were introduced between blocks. In addition, in order to analyze till which point the sequential effects would be comparable to random presentations, a random block, including also 180 trials, was introduced before the sequence learning task. Finally, participants were informed of the repeating pattern and they were asked to reproduce the sequence twice, without the visual stimuli, using the same response keys.
